# Jet Electroforming of High-Aspect-Ratio Microcomponents by Periodically Lifting a Necked-Entrance Through-Mask

**DOI:** 10.3390/mi15060753

**Published:** 2024-06-03

**Authors:** Yasai Zhang, Pingmei Ming, Xinmin Zhang, Xinchao Li, Lunxu Li, Zheng Yang

**Affiliations:** School of Mechanical and Power Engineering, Henan Polytechnic University, Jiaozuo 454000, China

**Keywords:** electroforming, high aspect ratio, electrodeposition, necked-entrance through-mask, jet electroforming

## Abstract

High-aspect-ratio micro- and mesoscale metallic components (HAR-MMMCs) can play some unique roles in quite a few application fields, but their cost-efficient fabrication is significantly difficult to accomplish. To address this issue, this study proposes a necked-entrance through-mask (NTM) periodically lifting electroforming technology with an impinging jet electrolyte supply. The effects of the size of the necked entrance of the through-mask and the jet speed of the electrolyte on electrodeposition behaviors, including the thickness distribution of the growing top surface, deposition defect formation, geometrical accuracy, and electrodeposition rate, are investigated numerically and experimentally. Ensuring an appropriate size of the necked entrance can effectively improve the uniformity of deposition thickness, while higher electrolyte flow velocities help enhance the density of the components under higher current densities, reducing the formation of deposition defects. It was shown that several precision HAR-MMMCs with an AR of 3.65 and a surface roughness (Ra) of down to 36 nm can be achieved simultaneously with a relatively high deposition rate of 3.6 μm/min and thickness variation as low as 1.4%. Due to the high current density and excellent mass transfer effects in the electroforming conditions, the successful electroforming of components with a Vickers microhardness of up to 520.5 HV was achieved. Mesoscale precision columns with circular and Y-shaped cross-sections were fabricated by using this modified through-mask movable electroforming process. The proposed NTM periodic lifting electroforming method is promisingly advantageous in fabricating precision HAR-MMMCs cost-efficiently.

## 1. Introduction

High-aspect-ratio micro- and mesoscale metallic components (HAR-MMMCs) can play some unique roles in quite a few application fields [1]. For instance, microcoils with aspect ratios (ARs) of more than 5 have enhanced the attraction of electromagnetic actuators and a reduced current and power consumption [2], tapered micropillars with ARs of 7 can provide efficient microfluidic device performance [3], and microelectrodes with ARs of 200 can minimize damage possibility in neural tissue penetration operations [4]. Therefore, the fabrication of HAR-MMMCs has always been one of the hottest research topics in the micromanufacturing field. To date, several micromanufacturing methods have been developed to produce HAR-MMMCs, and each of them has its own nature and advantages. These methods include electric discharge machining [5], mechanical microdrilling and micromilling [6], laser beam machining [7], electron beam machining [8], metal additive micromanufacturing processes [9], etc. However, the abovementioned methods either have great difficulty producing HAR-MMMCs in a large volume mode or have significant difficulty achieving the desired machining accuracy and material properties simultaneously.

Unlike the above HAR manufacturing methods, electroforming (EF) has a distinctive fabrication mode and possesses some unique potentials in manufacturing HAR-MMMCs [10,11,12]. EF forms metal materials by depositing metallic atoms layer upon layer, which are reduced from the metallic ions in an electrolyte driven by external electrical power. In such a way, the metal material is grown and thickened continually in a relatively low-temperature environment until the end metal article is achieved. In other words, EF manufactures metal articles in an additive manufacturing manner. Thereby, EF can form compact materials which can be easily tailored, and can fabricate low-stiffness precision microscale articles due to the very moderate material generation conditions and a low process temperature. According to whether a mask is used or not, electroforming can generally be divided into maskless electroforming (maskless EF) and through-mask electroforming (TM-EF). Maskless EF can theoretically fabricate HAR-MMMCs with a limitless height by continuously moving the anode or cathode during electrodeposition. For example, jet electroforming, a kind of typical maskless EF process [13,14,15], adopts an anodized high-speed jet electrolyte against the cathode as a tool to induce metal electrodeposition and the control material growth process. It has been verified that jet electroforming can form microcolumn structures at a considerably high current density (normally 100 A/dm^2^ or above), which means that the electrodeposition rate is very high, owing to the existence of high-speed jet electrolytes. Wang et al. used jet electroforming technology to obtain columnar microcomponents with an AR as high as 30 under the growth rate of 42 μm/min [13]. However, due to the unconstrained nature of the jet during electrodeposition, which induces serious stray current electrodeposition, the forming accuracy of jet electroforming is significantly low, making it very hard to manufacture precision articles [16]. Different from the maskless EF process, TM-EF has a much higher forming accuracy because it generates articles by filling well-defined through-mask cavities (i.e., mandrels) with the deposited material layer upon layer, and is generally dramatically accurate, since mandrels are normally created by short-wavelength lithography technology [17]. In theory, TM-EF can perfectly inversely replicate the cross-sectional shapes of mandrels. LIGA (Lithographie, Galvanoformung, Abformung) is a special TM-EF technique developed especially for fabricating precision HAR-MMMCs. It was reported that the AR of microcomponents manufactured by LIGA technology can be as high as more than 100 [18]. However, LIGA needs an expensive high-energy X-ray source and special photomask to prepare thick through-masks, limiting its wide commercial applications to a great extent. On the other hand, electroforming such HAR through-mask microcavities generally takes a very long time, even several weeks, because the mass transportation limitations inside HAR microcavities is seriously significant, greatly reducing the applied maximum current density [19,20,21]. In most industrial practices, the current densities applied to the LIGA electroforming process are below 2 A/dm^2^. To overcome these limitations, some lower-cost modified LIGA-like techniques such as UV-LIGA [22,23], Laser-LIGA [24], and ICP-RIE LIGA [25] have been developed by using conventional light sources or other methods, and photoresists to fabricate the masks. However, compared to LIGA technology, LIGA-like techniques cannot directly achieve HAR-MMMCs in a single step because the ARs of the through-masks they use are much lower. Although some researchers made some attempts to achieve HAR-MMMCs using multiple electroforming steps with a series of low-thickness through-masks, the electroforms fabricated have greatly reduced geometrical accuracy due to the unavoidable misalignment of the operations [22].

To more cost-efficiently electroform HAR-MMMCs, Zeng et al. [26] proposed a movable through-mask electroforming technology (movable TM-EF). Different from the conventional TM-EF technology, the through-mask in the movable TM-EF is lifted upwards constantly or periodically rather than being statically fixed on the cathode. This proposed process makes the manufacturing of HAR-MMMCs much easier, even when using lower-AR through-masks. In theory, it can fabricate micro- and meso-sized articles with a limitless AR. With this process, Zeng et al. successfully fabricated several HAR (≥3) structures, including E-shaped structures, columnar structures, and Y-shaped structures. However, this proposed movable TM-EF is inefficient in the electrodeposition rate, with an applied current density of 2 A/dm^2^ corresponding to a relatively low rate. Additionally, it does not show acceptable forming accuracy. The main reasons for these issues are likely poor mass transfer conditions and nonuniform current density distribution within the electrodeposition space [27,28,29,30]. Insulation shielding adjusts the electric field distribution between the anode and cathode by adding structures such as shielding plates outside the deposition area, thereby improving the uniformity of current density distribution on the cathode surface [28]. This study innovatively adopted this concept by installing a necked-entrance at the entrance of each through-mask cavity. This design reduces the edge effects of the electric field, enhancing the uniformity of current density distribution throughout the continuous deposition process. Therefore, this study proposes a modified movable TM-EF, specifically the periodically lifting necked-entrance TM-EF (NTM-EF). In the periodically lifting NTM-EF, the high-speed jet electrolyte transports the electrolyte into the cavities. In the following sections, verification studies will be conducted theoretically and experimentally, evaluating electrodeposition behaviors, surface morphology, dimensional accuracy, etc.

## 2. Simulation Analysis

### 2.1. Principle of Periodically Lifting NTM-EF

**Figure 1:**
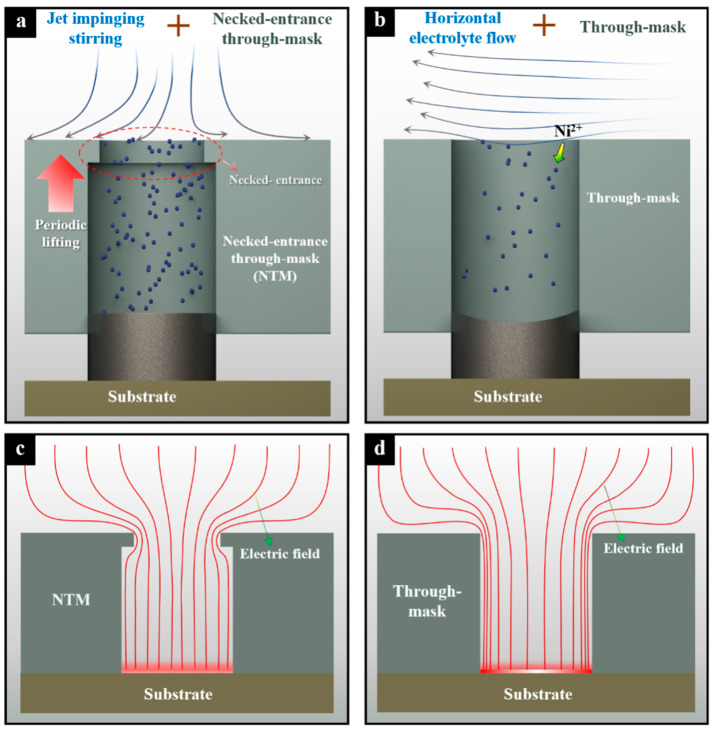
Schematical diagram of the two TM-EF technologies. (**a**) The periodically lifting NTM-EF with jet electrolyte; (**b**) The conventional movable TM-EF with horizontal electrolyte flow; (**c**) Current density distribution of NTM; (**d**) Current density distribution of conventional TM.

Figure 1a schematically shows the periodically lifting NTM-EF technology proposed in this study. Unlike the existing movable TM-EF shown in Figure 1b, this technology features a necked structure at the inlet entrance of each through-mask cavity to improve current density distribution within the cavities being filled, as shown in Figure 1c,d. Additionally, the proposed technology adopts a jet electrolyte against the through-mask cavity to accelerate the mass transport during electrodeposition. With these modifications, this study expects to achieve significantly enhanced deposition rate and forming accuracy of HAR-MMMCs using the periodic lifting NTM-EF [19,28,31].

**Figure 2:**
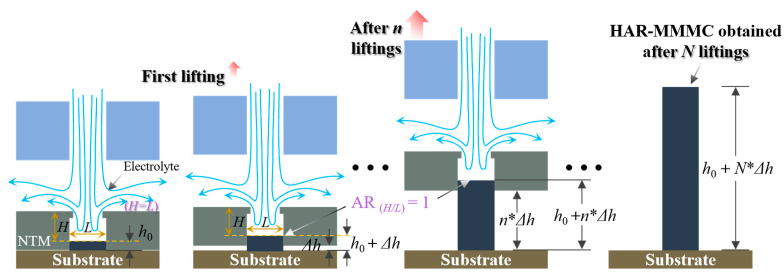
Workflows of the periodically lifting NTM-EF.

Figure 2 schematically shows the workflows of periodically lifting NTM-EF for fabricating the HAR-MMMCs. In this study, the growing surface is maintained at a relatively unchanged position inside the through-mask cavity. The aspect ratio (AR), defined as the ratio of the depth (H) to the diameter (L) of the through-mask cavity, is kept at approximately 1 by periodically lifting the NTM by a height of Δ*h* during deposition. In the initial stage of deposition, the bottom surface of the NTM is in close contact with the cathode substrate surface, with no relative movement between them. As the deposition process proceeds, metallic atoms are deposited inside the through-mask cavities. When the deposited metal grows to a thickness of *h*_0_, the NTM is lifted for the first time by a height of Δ*h*. When the deposited metal increases by another thickness of Δ*h*, the NTM is lifted again by Δ*h*. Thereafter, the NTM will be periodically lifted by Δ*h* whenever the deposited metal grows by Δ*h.* When the total thickness or height of the deposited article reaches the designed value, the deposition process stops. The parameter *h*_0_ is set to guide the deposited segment during the subsequent lifting process, ensuring proper alignment and stability. The small step size Δ*h* helps maintain stability during the lifting process, minimizing disruptions to the deposition and ensuring consistent quality. These parameters are critical for achieving uniform deposition and preventing detachment or misalignment of the deposited segment.

### 2.2. Simulation

#### 2.2.1. Models

**Figure 3:**
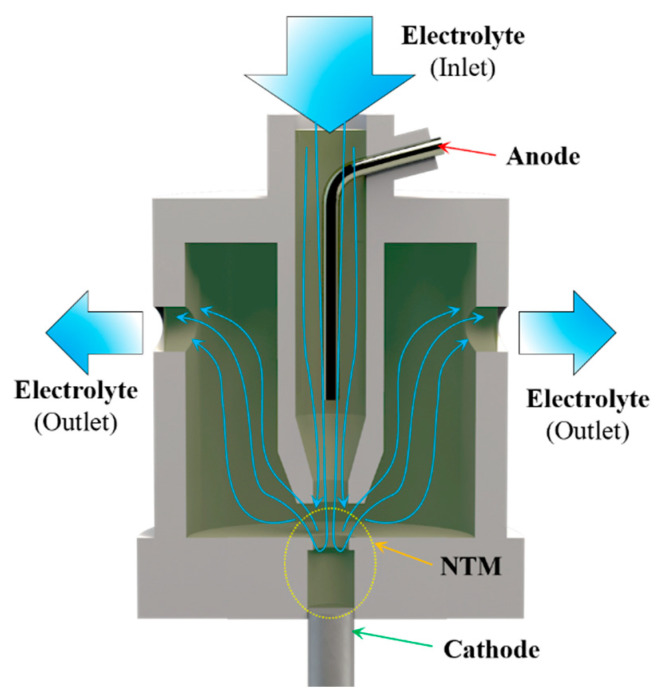
Schematic diagram of the core working components.

**Figure 4:**
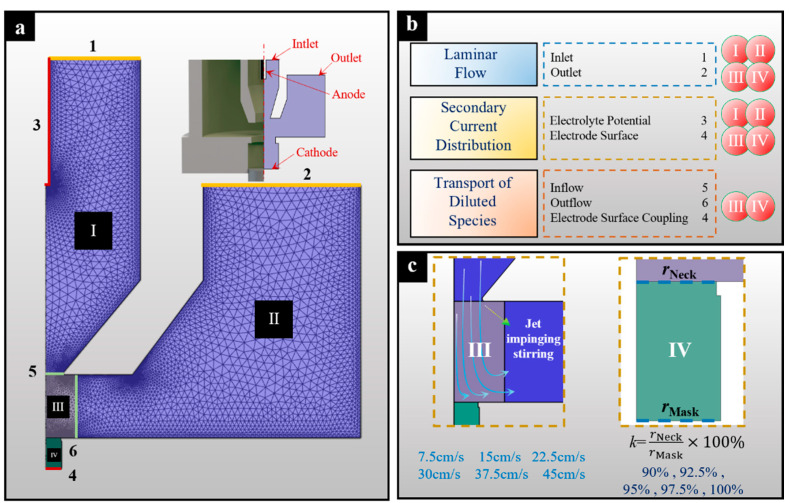
Numerical simulation model and its parameters definition. (**a**) Two-dimensional model; (**b**) Definition of boundaries of the model.; (**c**) Key initial condition parameters and the introduced factor.

To understand and optimize the distribution characteristics of mass transfer and current density, which determine the geometrical profile and growth rate of the deposits, numerical simulations were carried out. Figure 3 shows the core working components of the actual periodically lifting NTM-EF. Electrolyte is jetted against the entrance area of the through-mask from a circular hollow channel. It then flows out from the outlets located in the middle-upper part of a cylindrical tank after passing through the cavity. In this configuration, an electrically inert anode is fixed inside the hollow channel, located along the central line of the cylindrical tank. To assess the effectiveness of electroforming using a through-mask with a necked-entrance under jet conditions, a simulation model was established with a single mask cavity. Since the core working configuration is symmetrical, a two-dimensional physical model describing half of the configuration was developed to simplify the calculations, as shown in Figure 4.

#### 2.2.2. Simulations

The following assumptions are made for simulations to simplify the calculation without losing generality.

(1) The electrolyte is a continuous incompressible viscous fluid.
(2) The anode reaction is ignored, and the electrolyte potential is used as the anode boundary condition.
(3) The cathode reaction is nickel electrodeposition, ignoring cathode side reactions, and obeys Faraday’s law. The primary objective of the numerical simulations in this study is to investigate the effects of different necked-entrance parameters. These parameters affect the distribution of current density on the cathode surface and the mass transfer within the through-mask cavities. This study does not focus on the deposition efficiency of metal ions. Therefore, disregarding the cathodic side reactions is appropriate to meet the research objectives.
(4) The deposition surface of the microcomponent is at a stable position with an aspect ratio of the through-mask cavity of 1.

Since the electrochemical deposition process involves flow dynamics and electrochemistry, governing equations are required to solve the simplified model. The flow behavior of the electrolyte solution, as an incompressible continuous liquid, is governed by the Navier–Stokes equations, as shown below [32].

(1) ∇u=0

(2) $$\rho \left(\frac{\partial u}{\partial t}+u\cdot \nabla u\right)=-\nabla p+\nabla \left(\mu \left(\nabla u+{\left(\nabla u\right)}^{T}\right)\right)+F$$

where ρ stands for density, μ for dynamic viscosity, u for velocity, p for pressure, and F for surface tension.

The ion transport during the electrodeposition process involves changes in the solution’s composition, and its governing equations are as follows [33].

(3) $$\frac{\partial c}{\partial t}+\nabla \cdot J+u\cdot \nabla c=R$$

(4) $$J=-D\nabla c-zu_{m}Fc\nabla \varphi$$

where c stands for transported ion concentration, t for thermodynamic temperature, ∇ for gradient, J for material flux, u for velocity of the flowing fluid, R for nickel ion concentration sink, D for diffusion coefficient, F for Faraday constant, and ∇φ for potential gradient.

The electrical characteristics at the electrode interface are influenced by the action of ions in the solution, and their governing equations are as follows [28].

(5) $$j_{loc}=j_{0}\left(C_{R}\exp\left(\frac{\alpha_{a}F\eta }{RT}\right)-C_{O}\exp\left(\frac{\alpha_{c}F\eta }{RT}\right)\right)$$

(6) $$\eta =\phi_{s}-\phi_{l}-E_{eq}$$

where $j_{loc}$ stands for local current density, $j_{0}$ for exchange current density, $\alpha_{a}$ for anodic transfer coefficient, $\alpha_{c}$ for cathodic transfer coefficient [28], $C_{R}$ for reduction expression, $C_{O}$ for oxidation expression, F for Faraday constant, R for gas constant, T for thermodynamic temperature, $\Phi_{s}$ for electrode potential, $\Phi_{l}$ for electrolyte potential, and $E_{eq}$ for equilibrium potential of the electrodeposited metal.

A parameter, *k*, was introduced to numerically characterize the necking degree of the inlet of NTM:

(7) $$k=\frac{r_{Neck}}{r_{Mask}}\times 100\%$$

where $r_{Neck}$ stands for the radius of the necked-entrance, and $r_{Mask}$ for the radius of the normal NTM cavity.

To simplify the numerical simulation, a single through-mask cavity was selected, and its two-dimensional model is shown in Figure 4. The COMSOL Multiphysics (6.0) software was used to solve this model. The model is divided into four regions (I, II, III, and IV). Figure 4b shows the definitions of each region and boundary in the numerical simulation. In this simulation model, the secondary current distribution and laminar flow physics field are applied to Regions I, II, III, and IV. The transport of dilute species physics field is only applied to Regions III and IV. To improve the calculation accuracy, Regions III and IV were finely meshed because they operate on a microscale. The main data used for the numerical simulation model are listed in Table 1. Some key initial parameters and definitions are also presented in Figure 4b,c. In the simulations, the distribution of Ni^2+^ concentration in the deposition area under different inlet flow velocity conditions (*v*) was first analyzed. Then, the electric field distribution within the NTM cavity with different necked-entrance size was analyzed.

### 2.3. Simulation Results and Discussion

#### 2.3.1. Effect of Mass Transfer

**Figure 5:**
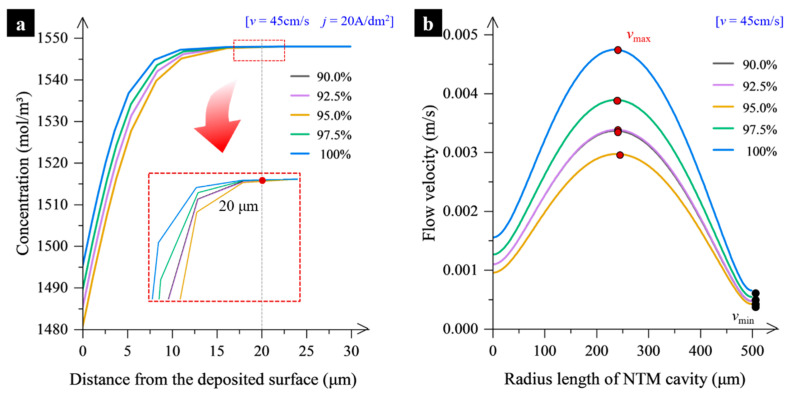
Change of the concentration distribution in the vicinity of the cathode surface and of the flow velocity distribution within the cavity. (**a**) Distribution of Ni^2+^ concentration within the electrolyte layer close to the cathode surface; (**b**) Distribution of the flow velocity along the radius of the NTM cavity at a distance of 20 μm away from the cathode surface.

In this study, *k* is an important characteristic parameter that affects the size of the mass exchange passage, determining the diffusion layer thickness and electrolyte flow velocity near the cathode surface. As shown in Figure 5a, the diffusion layer thickness remains almost constant at approximately 20 μm, despite changes in *k*. This means that the *k* value has little influence on the diffusion layer thickness. However, the *k* value significantly impacts the flow field distribution at a distance of 20 μm from the cathode surface along various distances from the NTM cavity axis, as shown in Figure 5b. Under the condition of electrolyte impingement, the flow field within the cavity fluctuates significantly. Generally, the flow velocity is smaller in the central region and near the cavity wall surface, but highest in the regions between the central part and the cavity wall. However, there are some differences when *k* is changed. When the necked-entrance is too large (i.e., *k* = 100%) or too small (i.e., *k* = 90%), the inflow and outflow of the electrolyte through the NTM cavity are significantly restricted. This leads to the generation of high-velocity regions and results in an extremely uneven flow field distribution within the cavity. However, when the necked-entrance size is appropriate (i.e., *k* = 95%), it greatly improves the uniformity of the flow field. This means that when *k* is selected to be 95%, the flow field distribution within the cavity is beneficial for the electroforming process.

**Figure 6:**
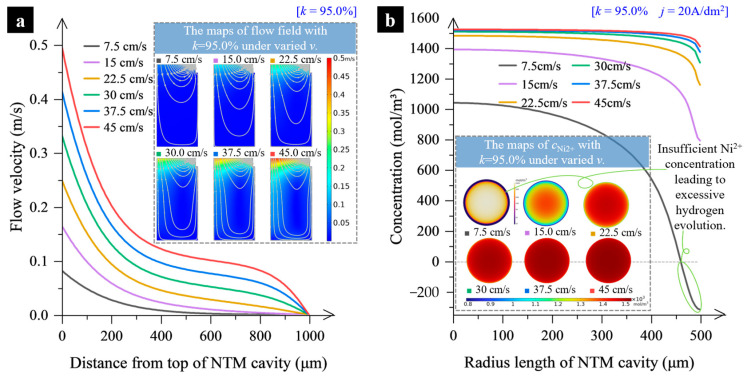
Change of the electrolyte flow velocity and the Ni^2+^ concentration distribution. (**a**) Distribution of the electrolyte flow velocity along the centerline of the NTM cavity; (**b**) Distribution of Ni^2+^ concentration within the electrolyte layer near the cathode surface.

Apart from *k,* the effect of the inlet flow velocity *v* on the mass transfer inside the cavity was also analyzed under the conditions of *k* = 95% and *j*_AVG_ = 20 A/dm^2^ (see Figure 6). It is shown that the velocity of the impinging electrolyte jet has a considerable impact on the flow field and mass transportation rate within the cavity. Generally, the higher the velocity *v*, the faster the electrolyte flows in the vicinity of the cathode surface, and the larger the mass transfer rate, as shown in Figure 6a. In addition, a higher jet-impinging velocity leads to faster electrolyte flow close to the cavity’s wall and a thinner diffusion layer. This results in a homogeneous Ni^2+^ concentration distribution, as shown in Figure 6b. These factors help enhance and homogenize the electrodeposition rate. Therefore, *v* = 45 cm/s is selected in this study.

#### 2.3.2. Electric Current Density Distribution

**Figure 7:**
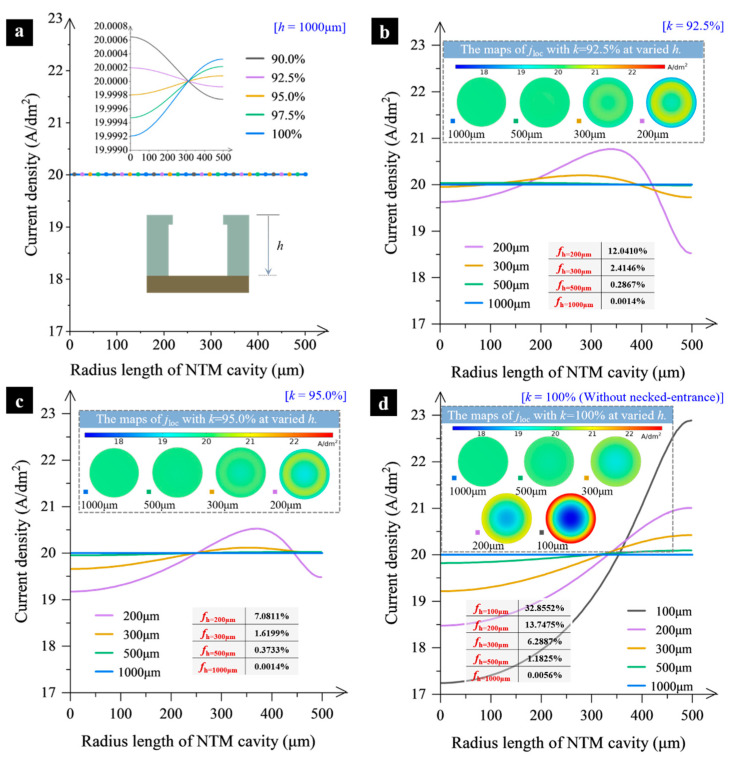
Change of current density distribution. (**a**) Current density distribution at the cross-section surface at the depth of *h* = 1000 μm; (**b**) Current density distribution with *k* = 92.5%; (**c**) Current density distribution with *k* = 95%; (**d**) Current density distribution with *k* = 100%.

The magnitude and distribution of the current density on the cathode surface are crucial factors determining the deposition speed and deposit thickness distribution uniformity. This study specifically analyzes the impact of changing the necked-entrance size on the current density distribution across the cavity’s cross-sections, as shown in Figure 7. These results were conducted with *j*_AVG_ = 20 A/dm^2^ and *v* = 45 cm/s.

Figure 7a indicates that current density distribution changes little in areas far from the NTM inlet. For example, at the cross-section of *h* = 1000 μm (depth measured from the top surface of the NTM), the change is minimal when the entrance size reduction is less than 10%. Further research shows that when the necked-entrance is 92.5% or 95% of the original size, the current density distribution is almost equal over the entire deposition surface. Based on this, the current density distribution at different distances *h* from the NTM top surface is further analyzed for *k* = 92.5%, 95%, and 100%, as shown in Figure 7b–d. The results show that when *k* = 92.5% and 95%, there are still some differences in the current density distribution at the cross-section close to the NTM cavity inlet. Compared with the scheme without necked-entrance, when *k* = 92.5% and 95%, the current density distribution at *h* = 500 μm still shows a high degree of uniformity. A deviation coefficient, *f*, is introduced to assess current density distribution uniformity, calculated using the formula *f* = (*j*_max_ − *j*_min_)/*j*_min_ × 100% [17,34]. Comparing the deviation coefficients for different *k* values shows that the proper necked-entrance size helps homogenize the current density distribution in the deposition area. *k* = 95% appears to be more effective in enhancing uniformity compared to *k* = 100% and 92.5%.

## 3. Experimental Study

### 3.1. Materials and Methods

**Figure 8:**
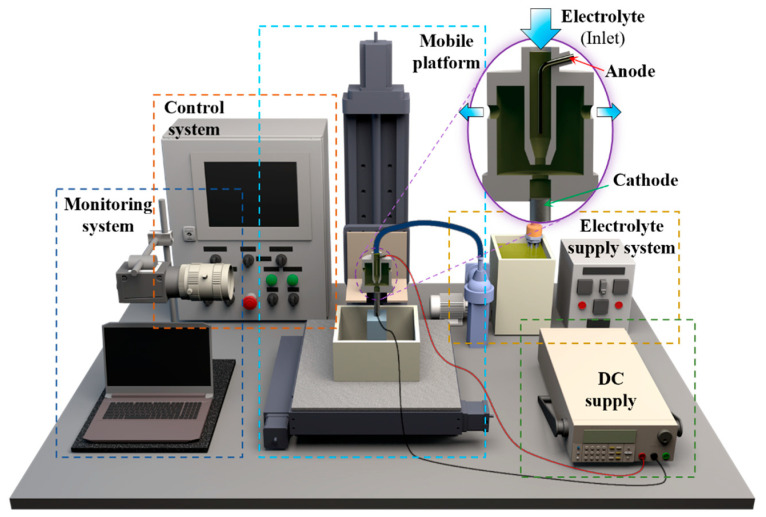
Schematic diagram of NTM-EF system.

Figure 8 shows the experimental setup specially developed for the NTM-EF technology. This setup mainly includes a mobile platform, an electrolyte supply system, a DC supply, a monitoring system, and a control system. The electrolyte compositions used were nickel sulfamate (500 g/L, AR, 98%), boric acid (30 g/L, AR, 98%), and wetting agent (0.15 g/L). The electrolyte temperature was maintained at 55 ± 1 °C. The pH of the electrolyte was maintained at 4 ± 0.2 by adding sulfamate acid or nickel carbonate. The electrolyte volume used in this study was relatively large, totaling 5 L. Due to the small amount of microcomponent deposition per experimental cycle, the inert anode minimally impacts the electrolyte pH. The pH was maintained at 4 ± 0.2 by adding nickel carbonate as needed after each experimental cycle. The electrodeposition lifting procedure was conducted using a self-built electroforming mobile platform. The power source employed for electrodeposition was a direct current (DC) power supply (ITECH, IT6122, Nanjing, China), while the monitoring system used was a charge-coupled device (CCD) microscope camera (ZQ, 616, Shanghai, China). Microhardness measurements of the components were carried out by a Vickers Hardness Tester (HV-1000, JITAI KEY, Beijing, China). Platinum was used as the anode material. To facilitate monitoring, both the nozzle and NTM shown in the enlarged view within the purple circle in Figure 8 are made from transparent organic glass. During the lifting process, strong adhesion between the deposited structure and the substrate is crucial to overcome frictional forces between the deposited segment and the mask cavity. This study used copper as the substrate material due to its strong adhesion to nickel, providing sufficient bonding strength for the deposition process.

In the experimentation, the parameter *h*_0_ represents the height of the deposited component within the through-mask cavity. If *h*_0_ is too large, it can result in excessive friction between the deposited component and the sidewalls of the through-mask cavity, hindering the lifting process. Conversely, if *h*_0_ is too small or if the fitting segment structure is not established, the alignment between the deposited section and the through-mask cavity will be insufficient. This inadequate alignment can cause the deposited section to detach from the through-mask cavity during the lifting process, leading to deposition failure. Therefore, *h*_0_/*L* = 0.2 is chosen as the fitting height. Additionally, if Δ*h* is too large, it can negatively impact the stability of the deposition process. Based on the specific conditions of the experimental setup, Δ*h* is selected to be 25 μm.

The deposited components were examined using a scanning electron microscope (Merlin Compact, Zeiss, Jena, Germany), a laser confocal microscope (Olympus, OLS5100, Tokyo, Japan) and a fully automatic image measuring instrument (COBEK, AM600CNC, Dongguan, China). Four hardness values were obtained from different samples to calculate the average value. The surface roughness and geometric dimensions were the averages of three measurements. The error bars shown in the figures represent the mean and standard deviation of multiple measurements.

### 3.2. Experimental Results and Discussion

**Figure 9:**
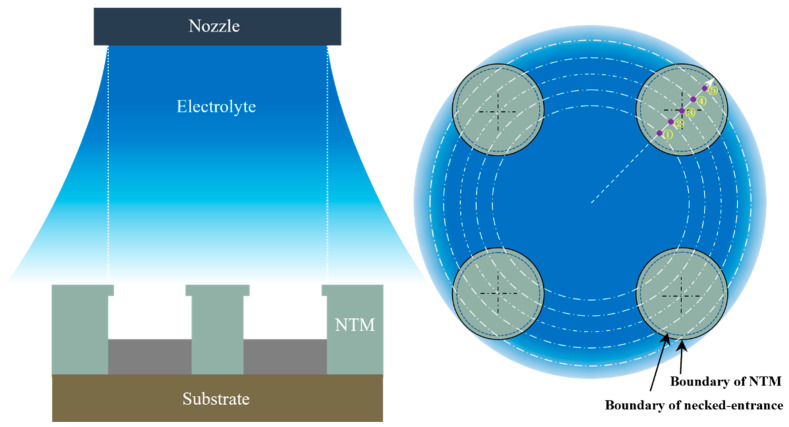
Schematic diagram of NTM-EF configuration.

As shown in Figure 9, the NTM has a small batch of mask cavities arranged on it, and the area corresponding to the nozzle aligns with these batch-arranged mask cavities. In subsequent experiments, five evenly spaced points along the white arrow pointing outward from the center of the jet region on the component were detected to evaluate the deposition effects. This was performed according to the layout shown in Figure 9.

**Figure 10:**
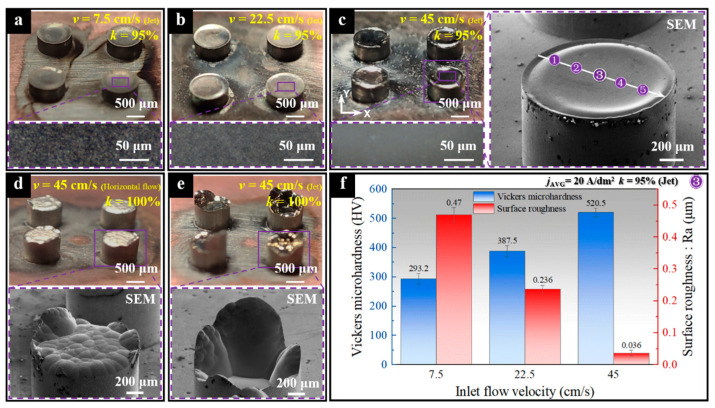
Electroforming effects of the columnar HAR-MMMCs under different conditions. (**a**) *v* = 7.5 cm/s (jet electrolyte), *k* = 95%; (**b**) *v* = 22.5 cm/s (jet electrolyte), *k* = 95%; (**c**) *v* = 45 cm/s (jet electrolyte), *k* = 95%; (**d**) *v* = 45 cm/s (horizontal electrolyte flow), *k* = 100%; (**e**) *v* = 45 cm/s (jet electrolyte), *k* = 100%; (**f**) Variation trend of Vickers microhardness and surface roughness of the components formed under different inlet flow velocities.

A study on the electroforming of columnar components with a feature size of 1000 μm was conducted with the mask stationary to verify the actual effect of the NTM in the electroforming process. Figure 10 shows images of the columnar components electroformed under five different conditions, all with the same deposition time (150 min) at *j*_AVG_ = 20 A/dm^2^. The top surface of the components fabricated with NTMs (with a necking degree of *k* = 95%) is smooth with few defects, regardless of the flow velocity. As the flow velocity increases, the top surface becomes smoother, more even, and has higher Vickers microhardness, as shown in Figure 10. For instance, at *v* = 45 cm/s, the top surface roughness at the center point position ③ of the deposited component is only 0.036 μm. At the same time, the Vickers microhardness at the same point of the component reached an impressive 520.5 HV. In comparison to the hardness range of nickel components obtained through conventional electrodeposition (266 HV–360 HV), this represents an increase ranging from 44.6% to 95.7%. There is a significant consistency in the quality of the deposition surface and Vickers microhardness under different flow velocities. In general, high current density can achieve smaller grain sizes, thereby enhancing the hardness of the deposited layer [35]. In this study, the current was kept constant while the electrolyte flow rate was varied. Typically, improving mass transfer, which increases the ion concentration level in the deposition area, can promote grain growth, which is generally unfavorable for enhancing microhardness. However, a significant increase in the density of the deposited layer was observed with higher electrolyte flow rates. Therefore, the changes in the performance of the deposited layer might be more related to the reduction in porosity and defects within the layer.

As shown in Figure 10d,e, when a through-mask without a necked-entrance is used for manufacturing, the surface of the component appears uneven, regardless of whether horizontal flow or jet flow is applied. These findings experimentally verified that an appropriate necked-entrance in the through-mask cavities improves formation accuracy and surface quality.

In this study, the electroforming effects at the center point position ③ of the component were analyzed and compared under conditions of *j*_AVG_ = 20 A/dm^2^ and *k* = 95% for different inlet flow velocities. As shown in Figure 10f, components electroformed at lower flow velocities exhibit lower microhardness and higher surface roughness. In contrast, components electroformed at higher flow velocities display higher microhardness and smaller surface roughness. A comparison reveals that at *v* = 7.5 cm/s, the component surfaces exhibit numerous pitting defects, while at *v* = 45 cm/s, the components appear dense with a mirror-like surface effect. According to previous studies [28], this trend may be attributed to poor mass transfer effects at lower electrolyte flow velocities. This leads to excessive hydrogen evolution in the deposition area, resulting in the formation of numerous pores within the components and adversely affecting microhardness. Conversely, higher electrolyte flow velocities provide more efficient mass transfer, reducing hydrogen evolution and resulting in denser components. Additionally, the use of higher current density promotes the formation of smaller grains, thereby enhancing the microhardness of the components.

**Figure 11:**
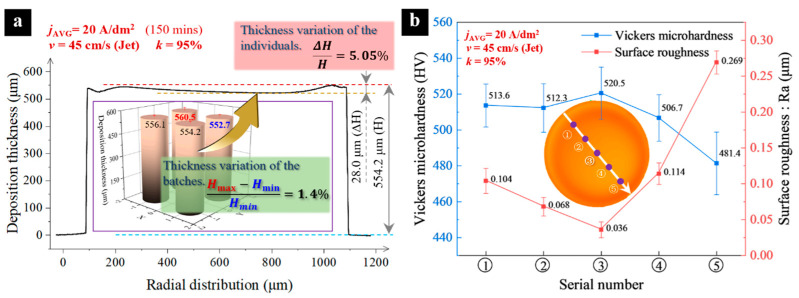
Electroforming effects of the components. (**a**) Height of the components; (**b**) Variation trend of Vickers microhardness and surface roughness of the components along the white arrow.

As shown in Figure 11a, when using an NTM with *k* = 95% under the experimental conditions of *v* = 45 cm/s jet flow, the thickness variation between the components manufactured in small batches is only 1.4%, ensuring stability in subsequent periodically lifting electroforming of components in small batches. In small-batch electroforming, considering the thickness distribution of an individual component along the direction of the white arrow, the thickness variation is 5.05%. Compared with the results reported in [26], the thickness variation is reduced by approximately 5% to 6%. Under this condition, the deposition rate of the components reached 3.6 μm/min ($\left(H-\Delta H∕2\right)∕150\;min$).

Figure 11b illustrates the distribution of Vickers microhardness and surface roughness at five evenly distributed points along the white arrow on the surface of the component. It can be observed that, from the center to the outer side of the jet, there is little difference in Vickers microhardness on the component’s surface. The microhardness at the outermost part of the component is slightly lower but still reaches 481.4 HV. Meanwhile, differences in surface roughness can be noted at different locations, with the minimum roughness at the center of the component and the maximum roughness at the outermost part, albeit only at 0.269 μm. Overall, the components manufactured using NTM-EF exhibit excellent performance.

**Figure 12:**
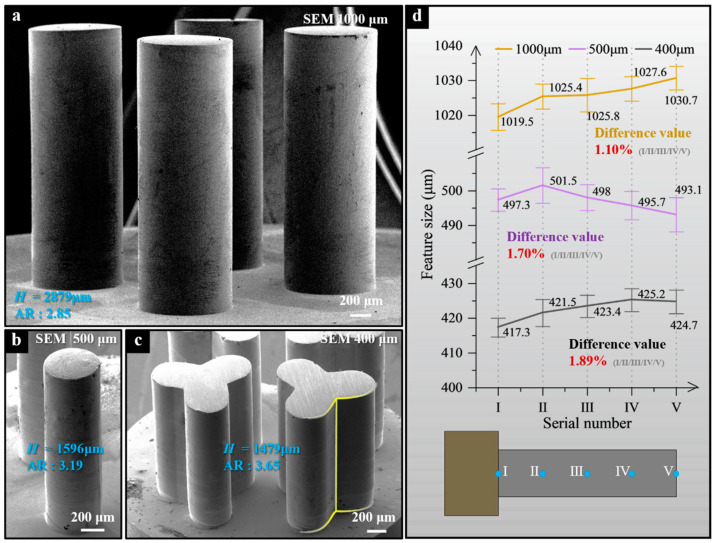
HAR-MMMCs after planarization. (**a**) Columnar HAR-MMMCs (designed diameter of 1000 μm); (**b**) Columnar HAR-MMMCs (designed diameter of 500 μm); (**c**) HAR-MMMCs with Y-shaped (designed line width of 400 μm); (**d**) The feature size variation of three scales of components.

To achieve higher micro- and mesoscale components, periodically lifting TMs were used during electroforming with the necked-entrance cavity molds. Figure 12a–c sequentially display HAR-MMMCs after planarization obtained through electroforming, each exhibiting varying dimensions and cross-sectional shapes. In these cases, highly precise structures, including those with complex cross-sections such as Y-shaped, can be successfully fabricated, as shown in Figure 12c. Additionally, Y-shaped components with complex cross-sections were produced, featuring straight sidewalls and clear boundaries. Upon inspection, their relative errors (the ratio of the absolute error to the standard design value) are within the desired range. Particularly noteworthy is the Y-shaped components with sharp boundary structures, achieving an aspect ratio of 3.65.

Figure 12d shows the analysis of feature dimensions at five uniformly sampled measurement points (I/II/III/IV/V) on the deposition height of components of different scales, revealing high forming precision in all three scales of components. Specifically, for the columnar component with a feature size of 1000 μm, the size difference is only 1.10%, while for the Y-shaped component with the AR of 3.65, the size difference is also merely 1.89%. This result demonstrates the significant potential of NTM-EF technology in achieving high-precision manufacturing of HAR-MMMCs.

## 4. Conclusions

To achieve high-speed and high-precision manufacturing of the high-aspect-ratio micro- and mesoscale metallic components (HAR-MMMCs), a periodically lifting necked-entrance through-mask electroforming (NTM-EF) process was proposed. The feasibility of the NTM-EF was examined numerically and experimentally. The following conclusions were drawn from the study.

(1) The periodically lifting NTM-EF process is feasible and effective for fabricating the precision HAR-MMMCs with a relatively high deposition rate.
(2) In the periodically lifting NTM-EF, the necking size of the through-mask cavity mold is crucial to achieve an evenly-distributed thickness of top-growing surface, and, generally, a slight necking of less than 10% is preferable.
(3) In the periodically lifting NTM-EF, a relatively higher speed of vertical impinging jet supply is necessary to quicken and homogenize mass transfer within the TM mold cavities.

## Figures and Tables

**Table 1:** The data used for simulations.

Parameter	Nomenclature	Value
Electrolyte temperature (°C)	*T*	55
Anodic charge transfer coefficient	*α*_a_	1.5
Cathodic charge transfer coefficient	*α*_c_	0.5
Cathodic current density (A/dm^2^)	*j*	20
Electrolyte conductivity (S/m)	*σ*	10
Concentration of Ni^2+^ (mol/m^3^)	*c*	1548
Electrolyte flow (cm/s)	*v*	7.5, 15, 22.5, 30, 37.5, 45
Electrolyte viscosity (Pa·s)	*μ*	1.2 × 10^−4^
Electrolyte density (kg/m^3^)	*ρ*	1400
Radius of through-mask cavity (μm)	*r_Mask_*	1000
*r_Neck_*/*r_Mask_* × 100%	*k*	90%, 92.5%, 95%, 97.5%, 100%

## Data Availability

The original contributions presented in the study are included in the article, further inquiries can be directed to the corresponding author.
